# A generic protocol for protein crystal dehydration using the HC1b humidity controller

**DOI:** 10.1107/S2059798316003065

**Published:** 2016-04-26

**Authors:** Carina M. C. Lobley, James Sandy, Juan Sanchez-Weatherby, Marco Mazzorana, Tobias Krojer, Radosław P. Nowak, Thomas L. Sorensen

**Affiliations:** a Diamond Light Source, Harwell Science and Innovation Campus, Didcot OX11 0DE, England; b Structural Genomics Consortium, University of Oxford, Roosevelt Drive, Headington, Oxford OX3 7DQ, England

**Keywords:** macromolecular crystallization, crystal dehydration, humidity control, cryoprotection, generic protocol, room temperature

## Abstract

Dehydration may change the crystal lattice and affect the mosaicity, resolution and quality of X-ray diffraction data. A dehydrating environment can be generated around a crystal in several ways with various degrees of precision and complexity. This study uses a high-precision crystal humidifier/dehumidifier to provide an airstream of known relative humidity in which the crystals are mounted: a precise yet hassle-free approach to altering crystal hydration. A protocol is introduced to assess the impact of crystal dehydration systematically applied to nine experimental crystal systems. In one case, that of glucose isomerase, dehydration triggering a change of space group from *I*222 to *P*2_1_2_1_2 was observed. This observation is supported by an extended study of the behaviour of the glucose isomerase crystal structure during crystal dehydration.

## Abstract 2

A generic protocol for investigating crystal dehydration is presented and tested with a set of protein crystal systems using the HC1b high-precision crystal humidifier/dehumidifier.

## 1. Introduction   

The process of obtaining X-ray diffraction-quality protein crystals can be fraught with difficulties. Large quantities of pure protein must be obtained and crystallization conditions must be determined through an empirical process (see McPherson, 1999 ▸ and references therein). Typically, any crystal obtained will then be removed from the mother liquor in which it was grown, exposed to a cryoprotectant and flash-cooled in liquid nitrogen (Garman & Schneider, 1997 ▸). These challenges navigated, the protein crystal is exposed to X-rays, usually using the intense X-ray beams available at a synchrotron source. Ideally, this process will result in the collection of useful diffraction data and the protein structure can be solved. More often the crystals will diffract X-rays weakly, with high mosaicity or anisotropy, or worse, not at all. In these cases it is not possible to collect a suitable X-ray diffraction data set.

There are two main routes to progress from initial crystal leads or poorly diffracting crystals to crystals from which an X-ray diffraction data set can be collected: pre-crystallization optimization of the protein and/or crystallization conditions such that a better crystal is obtained, or post-crystallization optimization to work with the crystals already obtained and improve their quality (Heras & Martin, 2005 ▸; Newman, 2006 ▸). Two important post-crystallization approaches can be pursued concurrently: the cryoprotection strategy (Garman, 1999 ▸) and crystal dehydration (Einstein, 1961 ▸; Huxley & Kendrew, 1953 ▸; Pickford *et al.*, 1993 ▸; Kiefersauer *et al.*, 2000 ▸, 2014 ▸; Sjögren *et al.*, 2002 ▸; Sanchez-Weatherby *et al.*, 2009 ▸; Bowler *et al.*, 2015 ▸).

Crystal dehydration and cryoprotection are intimately linked. Crystals are grown in small drops of mother liquor isolated from the ambient environment. Accessing the crystallization drop initiates crystal dehydration, which is then compounded by the application of a cryoprotecting solution, usually with a lower relative humidity than the initial crystallization condition. Given the low volumes routinely used for automated crystallization (∼200 nl), harvesting a crystal for cryocrystallography can have a significant, uncontrolled impact on the crystal hydration state (Farley *et al.*, 2014 ▸).

The humidity-controlled experiment provides a method to systematically explore the impact of relative humidity on the diffraction properties of the crystal (Kiefersauer *et al.*, 2000 ▸, 2014 ▸; Sanchez-Weatherby *et al.*, 2009 ▸; Douangamath *et al.*, 2013 ▸; Bowler *et al.*, 2015 ▸). Using the HC1b (Arinax; Sanchez-Weatherby *et al.*, 2009 ▸; Russi *et al.*, 2011 ▸; Bowler *et al.*, 2015 ▸), the crystal is removed from mother liquor using a mesh and is held in a stream of air at the same relative humidity as the mother liquor (Wheeler *et al.*, 2012 ▸). To investigate the impact of the cryoprotection strategy, the crystal is mounted naked; that is, with no surrounding mother liquor (Pellegrini *et al.*, 2011 ▸). In this state, the diffraction quality at 295 K can be tested. In addition, the crystal can be flash-cooled, with no additional chemical cryoprotectant, to test for the potential formation of hexagonal ice and protein crystal diffraction at 100 K. This provides an indication of the fundamental diffraction quality of the sample. To study the impact of crystal dehydration, the relative humidity of the sample can be lowered systematically. The quality of the X-ray diffraction from dehydrated samples can be tested directly at 295 K or after cryocooling at 100 K.

Dehydration is usually carried out as a last resort for targets that have proven to be particularly challenging. There are a number of structures that have been solved after crystal dehydration (for a recent review, see Russo Krauss *et al.*, 2012 ▸; for more recent examples, see Hu *et al.*, 2011 ▸; Oliete *et al.*, 2013 ▸; Malinauskaite *et al.*, 2014 ▸; Hellmich *et al.*, 2014 ▸). Each has been studied as a unique target, resulting in improved diffraction and a higher resolution X-ray structure. Consequently, it is unclear whether a single dehydration protocol can be applied to all targets for dehydration. Additionally, with only successful cases reported in the literature, the frequency with which dehydration or 295 K data collection may assist with obtaining useful X-ray diffraction data is unclear.

**Figure 1:**
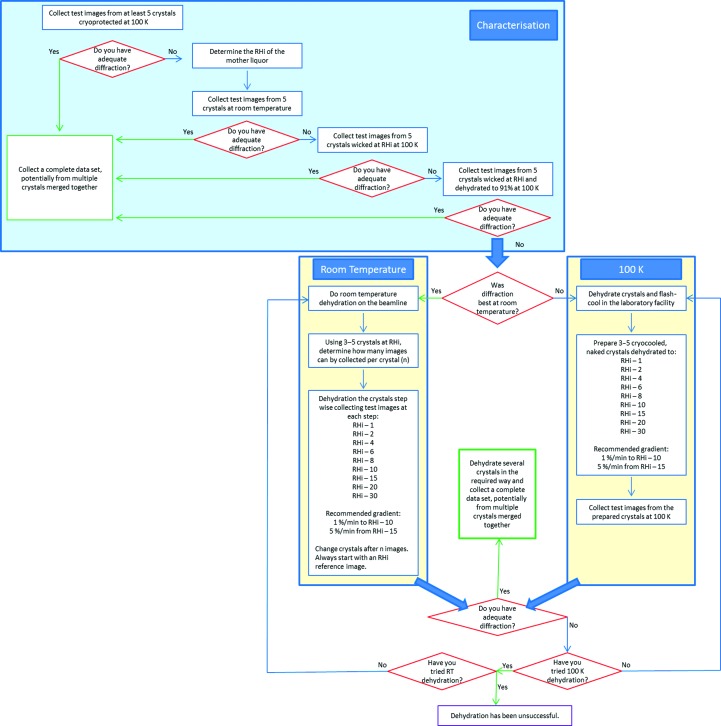
The generic dehydration protocol. The initial phase in the generic dehydration protocol is characterization of the crystals to be dehydrated (blue). This should be followed by both a 295 and 100 K investigation of the impact of crystal dehydration (yellow).

We present a generic method to work with crystal dehydration using the HC1b (Fig. 1 ▸). After determination of the initial relative humidity (RHi), crystal diffraction needs to be tested at 295 K and using cryocooled, naked crystals along with crystals prepared for a standard 100 K experiment. This initial characterization demonstrates the potential of the crystals. Subsequently, this protocol provides two complementary routes for the thorough investigation of the effects of dehydration: (i) multiple crystals can be prepared in each of a suggested nine conditions and cryocooled prior to beamtime or (ii) individual crystals can be dehydrated at the beamline and diffraction images can be collected and analysed during the course of dehydration. The former route has the advantage of using beamtime more efficiently, since the dehydration is performed in advance of beamtime and multiple crystals can be studied in each condition. The latter option gives the advantage of being able to work at 295 or 100 K for data collection and to analyse the X-ray diffraction data as the experiment progresses, enabling the experiment to immediately be adjusted to fit the sample.

Through the application of our protocol to nine experimental targets, we assess the general applicability of the method proposed and address the question of how frequently dehydration or 295 K data collection could improve the quality of diffraction data from macromolecular crystals.

## 2. Materials and methods   

### 2.1. Crystallization of target proteins   

The nine soluble protein targets used in this work were provided by Diamond Light Source or the Structural Genomics Consortium (SGC), Oxford, or were purchased from commercial suppliers.

The JmjC domain of human histone 3 lysine-specific demethylase 3B (JMJD1B), purified at the SGC (http://www.thesgc.org/structures/4C8D), was crystallized by mixing 100 nl 20 mg ml^−1^ protein solution in 10 m*M* HEPES pH 7.5, 500 m*M* NaCl, 5%(*v*/*v*) glycerol, 0.5 m*M* TCEP, 2 m*M* *N*-oxalylglycine, 6 m*M* MnCl_2_ with 50 nl reservoir solution consisting of 0.1 *M* bis-tris pH 5.5, 0.1 *M* ammonium acetate, 27%(*w*/*v*) PEG 3350. Cuboid-shaped crystals (100 × 80 × 50 µm) appeared within several days from sitting-drop plates at 293 K.

The bromodomain of human BAZ2B was purified as described previously (Ferguson *et al.*, 2013 ▸). It was crystallized by mixing 75 nl 10.8 mg ml^−1^ protein solution in 10 m*M* HEPES pH 7.5, 500 m*M* NaCl, 5%(*v*/*v*) glycerol with 75 nl reservoir solution consisting of 25–35%(*v*/*v*) PEG 600, 0.1 *M* MES pH 6.3, 0–8%(*v*/*v*) glycerol. Pyramid-shaped crystals (100 × 100 × 100 µm) appeared within several days from sitting-drop plates at 277 K.

The tandem tudor domain of human JMJD2A (Tudor), which was purified at the SGC (http://www.thesgc.org), was crystallized by mixing 50 nl 11.5 mg ml^−1^ protein solution in 20 m*M* HEPES pH 7.5, 500 m*M* NaCl, 5%(*v*/*v*) glycerol with 100 nl reservoir solution consisting of 0.1 *M* Tris pH 8.5, 1.6–2 *M* ammonium sulfate. Cubic crystals (50 × 50 × 50 µm) appeared within several days from sitting-drop plates at 293 K.

Human JMJD2D, which was purified at the SGC (http://www.thesgc.org), was crystallized by mixing 2 µl 12 mg ml^−1^ protein solution with 2 µl reservoir solution consisting of 30%(*w*/*v*) PEG 3350, 0.1 *M* HEPES pH 7.0, 0.15 *M* ammonium sulfate. Bipyramidal crystals (100 × 100 × 100 µm) appeared in 2 days from sitting-drop plates at 293 K.

Human MINA53 construct Ala26–Val464, which was purified at the SGC (http://www.thesgc.org), was crystallized by mixing 21.7 mg ml^−1^ protein solution in 10 m*M* HEPES pH 7.5, 500 m*M* NaCl, 5%(*v*/*v*) glycerol, 0.5 m*M* TCEP with reservoir solution consisting of 0.1 *M* HEPES pH 7.5, 18% PEG 3350, 5 m*M* CdCl_2_ to give a total drop volume of 150 nl using 2:1, 1:1 and 1:2 protein:precipitant ratios. Hexagonal crystals (75 × 75 × 75 µm) appeared overnight from sitting-drop plates at 293 K.

The second bromodomain of human pleckstrin homology domain interacting protein [PHIP(2)] was purified as described previously (Filippakopoulos *et al.*, 2012 ▸). It was crystallized by mixing 2 µl 12 mg ml^−1^ protein solution with 2 µl reservoir solution consisting of 6%(*w*/*v*) PEG 3350, 0.1 *M* sodium acetate pH 5.6. Rod-like crystal clusters (100 × 50 × 50 µm) appeared in one week from sitting-drop plates at 277 K.

The catalytic domain of human tryptophan hydroxylase 2 (TPH2) was purified at the SGC (http://www.thesgc.org/structures/4V06). TPH2 was crystallized by mixing 100 nl 13.4 mg ml^−1^ protein solution in 25 m*M* HEPES pH 7.5, 250 m*M* NaCl, 5%(*v*/*v*) glycerol with 50 nl reservoir solution consisting of 0.1 *M* sodium citrate pH 5.5, 14%(*w*/*v*) PEG 3350, 10%(*v*/*v*) ethylene glycol. Plate-like crystals (100 × 80 × 20 µm) appeared overnight from sitting-drop plates at 293 K.

Proteinase K from *Tritirachium album* (Sigma–Aldrich, catalogue No. P2308) was crystallized by mixing 1 µl 25 mg ml^−1^ protein solution in 25 m*M* HEPES pH 7.0, 100 µ*M* PMSF with 1 µl reservoir solution consisting of 0.1 *M* bis-tris pH 5.5, 0.65 *M* LiCl. Bipyramidal crystals (100 × 100 × 200 µm) appeared overnight from sitting-drop plates at 293 K.

Glucose isomerase from *Streptomyces rubiginosus* (Hampton Research, catalogue No. HR7-102) was dialysed into 25 m*M* Tris–HCl pH 7.5 and was then crystallized by mixing 2 µl 25 mg ml^−1^ protein solution with 2 µl reservoir solution consisting of 10%(*w*/*v*) PEG 400, 20%(*w*/*v*) glucose, 50 m*M* MnCl_2_, 0.1 *M* HEPES pH 7.0. Rhombic dodecahedral crystals (100 × 100 × 30 µm) appeared within 2 d from sitting-drop plates at 293 K.

### 2.2. Generic dehydration protocol   

#### 2.2.1. Determination of initial relative humidity   

The RHi of each mother liquor was determined by a method similar to that published elsewhere (Wheeler *et al.*, 2012 ▸). A 300–500 µm nylon loop containing mother liquor was mounted in the HC1b airstream. The size of the drop was monitored using edge-detection routines in the image-analysis software provided by the EMBL HC1 control software. The humidity provided by the HC1b was adjusted until the drop size remained constant, indicating that the RHi had been reached. Once the RHi had been determined then a second sample of mother liquor was mounted to qualify the initial measurements.

#### 2.2.2. Initial crystal characterization   

Crystals for initial characterization were prepared in the laboratory at 295 K and ambient humidity. Crystals were exposed to the humidity of the laboratory for less than 3 s during harvesting.

Five crystals of each target were prepared by harvesting the crystals and flash-cooling in liquid nitrogen after a 30 s to 1 min sweep though 30%(*v*/*v*) ethylene glycol [proteinase K, glucose isomerase, TPH2, Tudor, JMJD1B, JMJD2D and PHIP(2)] or 30%(*v*/*v*) glycerol (BAZ2B and MINA53) made up in mother liquor.

Five crystals of each target were prepared by mounting on a mesh and wicking the crystal dry before flash-cooling in liquid nitrogen. In all cases, wicking the crystal was carried out with the crystal at RHi in the HC1 airstream. Wicking was achieved using a paper wick (MiTeGen) to touch the underside of the mesh on which the crystal was mounted. By touching the mesh and not the crystal, it is possible to withdraw the mother liquor, leaving the crystal with no visible liquid.

Five crystals of each target were prepared by mounting on a mesh, wicking the crystal dry and dehydrating to a RH of 91% before flash-cooling in liquid nitrogen. The RH of 91% is the average of the RH of 30% ethylene glycol and 30% glycerol and therefore reflects chemically cryoprotected conditions.

Five crystals of each target were prepared by mounting on a mesh on the beamline, wicking dry and collecting data at 295 K.

Data collection consisted of collecting three images per crystal with a φ separation of 45°. Oscillation images were collected using low transmission (giving approximately 8 × 10^11^ photons s^−1^) with 1° oscillation over 1 s. Data collection was carried out on beamline I02 at Diamond Light Source using the standard energy of 12 658 eV. This strategy was chosen to increase the chance of successful indexing. Data were indexed, unit-cell parameters were established and the mosaicity was estimated using *MOSFLM* (Leslie, 2006 ▸; Powell *et al.*, 2013 ▸).

#### 2.2.3. Diffraction from cryocooled, dehydrated crystals   

**Figure 2:**
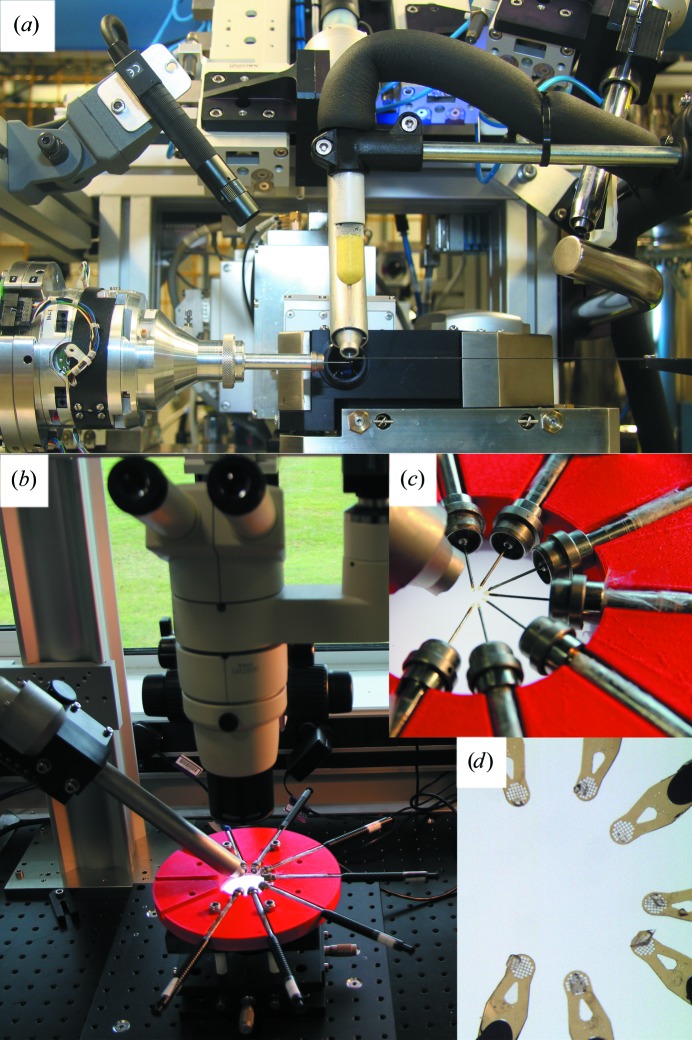
The HC1b at Diamond Light Source. (*a*) shows the HC1b positioned in place of the cryostream on beamline I02 at Diamond Light Source. (*b*) shows the HC1b in the ‘high-throughput’ setting in the laboratory facility at Diamond Light Source, enabling crystals to be prepared in advance of beamtime. The multi-sample holder developed by Diamond Light Source can clearly be seen in red. (*c*) shows a close-up of the arrangement of meshes mounted in the humid airstream. (*d*) shows a view of seven wicked crystals on meshes as observed using the HC1 software.

The HC1b was used in the laboratory facility at Diamond Light Source (Figs. 2 ▸ *b*, 2 ▸ *c* and 2 ▸ *d*). Crystals were mounted on meshes and were wicked dry. A sample holder has been developed whereby it is possible to prepare up to seven crystals in parallel. The multi-sample holder is of a circular design with a central hole cut out to allow illumination of the samples from a flat LED panel mounted underneath (Fig. 2 ▸ *b*). Seven crystal wands, each holding a mesh, are positioned such that the mesh is in the central hole (Fig. 2 ▸ *c*). The HC1 nozzle approaches the centre of the hole at approximately a 30° angle, exposing all seven meshes to the humid airstream. Directly above the sample holder a microscope is installed to allow the user to see their samples directly, or they can be viewed using the HC1 control software (Fig. 2 ▸ *d*).

In this case, four crystals were prepared in parallel at 1, 2, 4, 6, 8 and 10% below the RHi using a dehydration rate of 1% per minute with a final wait of 3–5 min. Four crystals were prepared at 15, 20 and 30% below the RHi using a dehydration rate of 5% per minute with a final wait of 3–5 min.

Data were collected as for the initial characterization.

#### 2.2.4. Diffraction from room-temperature, dehydrated crystals   

The HC1b was used on beamline I02 at Diamond Light Source (Fig. 2 ▸ *a*). The temperature at the beamline was 295 K, with a humidity level of 20%. Crystals were exposed to the humidity of the beamline for less than 3 s while they were mounted on meshes. They were wicked dry at RHi in the HC1 airstream as for samples prepared in the laboratory. The radiation sensitivity was tested by taking up to ten diffraction images of the crystal using an oscillation of 1° over 1 s with low transmission (giving approximately 8 × 10^11^ photons s^−1^).

The observed results at 100 K were used to guide the 295 K experiment: in cases where dehydration had little effect on the crystal fewer more coarse steps were used at 295 K, whilst those samples where diffraction was lost were only tested to the point of loss at 295 K. Dehydration up to and including 10% below the RHi was carried out at 1% per minute, while dehydration beyond this point used a gradient of 5% per minute. In each case there was a final wait of 3–5 min. Dehydration protocols were managed using the HC1 interface within the *Generic Data Acquisition* (*GDA*) software available on the beamlines at Diamond Light Source (http://www.opengda.org; http://www.diamond.ac.uk/Beamlines/Mx/I02/I02-Manual/Using-New-GDA/The-HC1-Perspective.html).

Data were collected as for the initial characterization and for cryocooled crystals.

### 2.3. Extended dehydration study of glucose isomerase   

An extended study of glucose isomerase crystals was carried out using the HC1b on beamline I02 at Diamond Light Source. For this work, a single 14-day-old batch of glucose isomerase crystals was used. The RHi for this crystallization was determined to be 96%.

A number of dehydration protocols were carried out in triplicate (Table 1 ▸), primarily focused on investigating the impact of the rate of dehydration: slow (1% per minute), medium (2% per minute) and fast (5% per minute). In addition, abrupt changes to the RH of the crystal were induced by mounting at different humidities, with the intention of determining the exact point where change is induced in the crystals. Crystals were mounted using meshes and were wicked to remove all traces of mother liquor. After analysis at 295 K, the crystal was translated such that the X-ray beam would illuminate a new part of the crystal. The HC1b and cryojet were swapped to flash-cool the crystal and data were collected at 100 K.

Data for indexing were collected as for the initial characterization, with the exceptions that the beam size was reduced to 50 × 28 µm (from 85 × 28 µm) using the beam-collimating slits, that the oscillation per image was reduced to 0.5° and that the transmission was tenfold lower (giving approximately 8 × 10^10^ photons s^−1^). Data were indexed using *EDNA* (Incardona *et al.*, 2009 ▸) as implemented in the automated software pipeline at Diamond Light Source.

Full data collection was carried out using the starting angle and recommended oscillation angle from *EDNA* using either 0.04 or 0.05 s exposure times and an increased flux (approximately 2 × 10^11^ photons s^−1^).

Data were processed using *xia*2 (Winter, 2010 ▸; Winter *et al.*, 2013 ▸) and *DIALS*, and the data were cut to 2 Å resolution for consistency across all data sets. Molecular replacement was carried out with a glucose isomerase structure (PDB entry 1mnz; E. Nowak, S. Panjikar & P. A. Tucker, unpublished work) as a search model using *MOLREP* (Vagin & Teplyakov, 2010 ▸) within the *CCP*4 package of programs (Winn *et al.*, 2011 ▸). The resultant structure files were refined using rigid-body followed by restrained refinement using *REFMAC* (Murshudov *et al.*, 2011 ▸). Rebuilding, placement of ligands and water checking were carried out in *Coot* (Emsley *et al.*, 2010 ▸) followed by iterative rounds of refinement and rebuilding.

## 3. Results   

### 3.1. Initial relative humidity   

The initial relative humidity for the dehydration experiments was determined for each sample by measuring that of their mother liquor (Table 2 ▸). Crystallization solutions with low-concentration salts or high molecular weight PEG solutions [proteinase K, Tudor, JMJD1B, JMJD2D, PHIP(2) and MINA53] tend to require a higher RH to be in equilibrium with the humid airstream, *i.e.* drops of these solutions remained a constant size when placed in the humid airstream. Conversely, solutions with high salt concentration or low-molecular-weight PEG solutions (glucose isomerase, TPH2 and BAZ2B) are stable at lower RH. Table 1 ▸ shows that our empirical observations are in good agreement with theoretical values described in previous systematic studies (Wheeler *et al.*, 2012 ▸).

Typically, cryoprotecting solutions equilibrate with the humid airstream at a lower RH than those which do not cryoprotect. In principle, dehydrating a crystal increases the precipitant concentration within the solvent channels, leading to cryoprotection. In order to be able to assess the validity of using only dehydration as a cryoprotecting process, the relative humidity of two well established cryoprotecting solutions was determined. The relative humidities of 30% glycerol and 30% ethylene glycol were measured to be 92 and 90%, respectively. For this reason, the average relative humidity of 91% was subsequently used in further experiments to test the validity of relative humidity as a mimic of chemical cryo­protection.

### 3.2. Application of the generic dehydration protocol   

**Figure 3:**
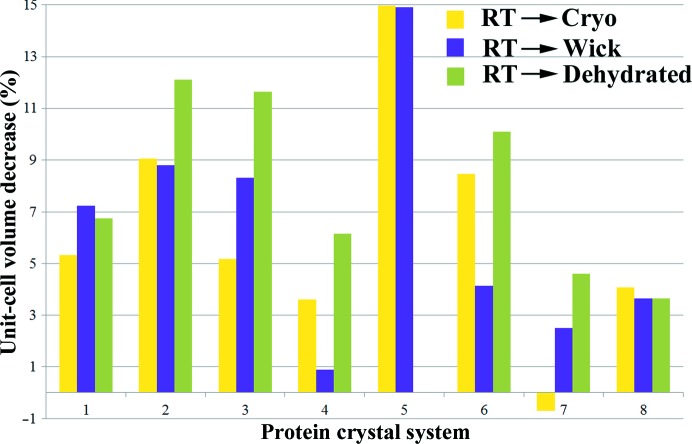
Changes in the unit-cell volume in initial crystal characterization. During initial characterization the unit-cell volume change was monitored with respect to the initial 295 K unit cell. The unit-cell volume decrease upon cryoprotection is shown in yellow, the unit-cell volume decrease upon cryocooling naked crystals is presented in purple and the unit-cell volume decrease upon cryocooling naked crystals at a RH of 91% is shown in green. Each set of bars represents one protein crystal system: 1, BAZ2B; 2, Tudor; 3, JMJD2D; 4, MINA53; 5, PHIP(2); 6, TPH2; 7, proteinase K; 8, glucose isomerase (*I*222). PHIP(2) did not tolerate dehydration, so there is no result for this system. *P*222 glucose isomerase did not occur at 295 K so only *I*222 glucose isomerase is represented.

To assess the impact of dehydration on each crystal system, it is necessary to compare the results of all three sections of the generic dehydration protocol; namely, the initial crystal characterization, the dehydration and cryocooling of crystals before diffraction testing at 100 K and the dehydration of crystals before diffraction testing at 295 K (Fig. 1 ▸). The results are summarized in Fig. 3 ▸ and are presented in full in Supplementary Tables S1–S8.

Throughout these discussions the percentage unit-cell volume change is used as an indicator of lattice change. It has been reported previously that in general a 2% volume change is the upper limit that MIR phasing can tolerate (Garman & Murray, 2003 ▸), thus suggesting that at least this magnitude of change would be expected upon dehydration if it is to significantly alter the structural arrangement of the crystal lattice.

#### 3.2.1. JMJD1B   

JMJD1B gave no indexable diffraction in the initial crystal characterization. As a result of dehydration both with cryocooling and at 295 K, those few reflections observed during characterization were no longer present. This is an example where dehydration was unable to improve crystals with very low intrinsic X-ray diffraction power.

#### 3.2.2. BAZ2B   

**Figure 4:**
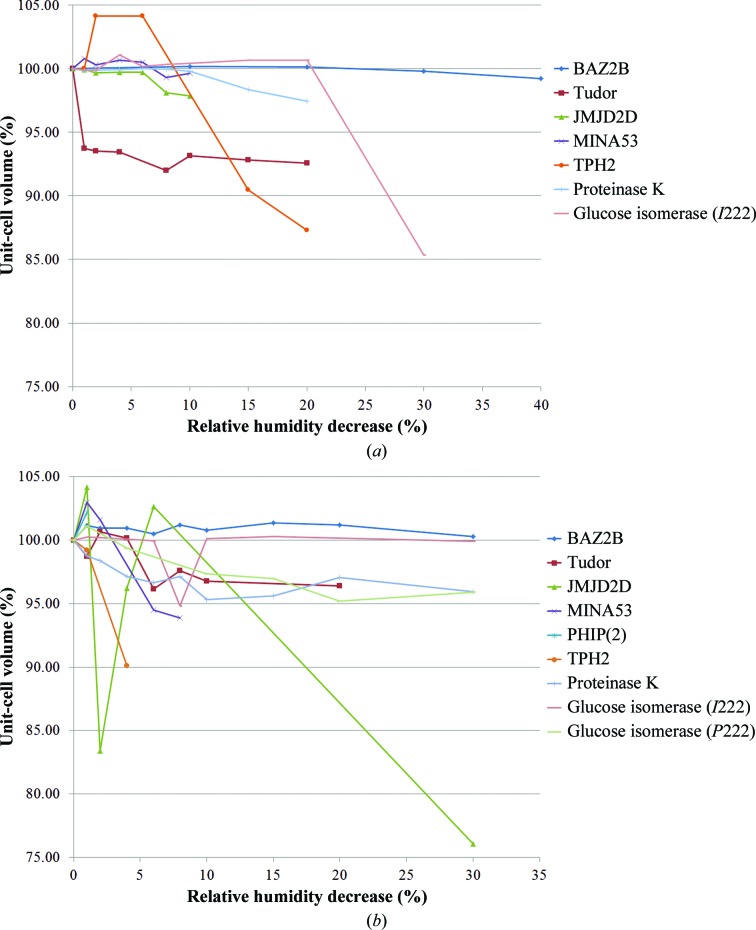
Unit-cell volume changes with dehydration. (*a*) shows the changes in unit-cell volume caused by dehydration for data collected at 100 K, while (*b*) is for data collected at 295 K. In both cases, the line for the given protein stops at the relative-humidity decrease that gave the last indexable data.

In the initial characterization, the *C*222_1_ BAZ2B crystals were observed to undergo a unit-cell contraction compared with the 295 K sample of 5.3% when cryocooled, 7.2% when wicked and cryocooled and 6.7% when dehydrated to a RH of 91% and cryocooled (Fig. 3 ▸, Supplementary Table S1). Dehydration with data collection at 100 K showed no substantial change in the unit-cell volume with respect to the cryocooled unit cell. Dehydration followed by data collection at 295 K showed no substantial change in the unit-cell volume with respect to the 295 K unit cell (Fig. 4 ▸, Supplementary Table S1). X-ray diffraction was observed to a resolution of 2 Å or better and the mosaicity was lowest in the 295 K crystals.

BAZ2B crystallized with a compact, stable lattice. The only lattice change that occurred was in response to thermal contraction during cryocooling, and the system was not susceptible to dehydration. There are numerous structures of BAZ2B available in the Protein Data Bank (PDB), all of which were obtained using diffraction to higher than 2 Å resolution, that have very similar unit-cell dimensions to those presented here, indicating that this sample is commensurate with other samples previously studied.

#### 3.2.3. Tudor   

Tudor crystals were observed in space group *I*23. In the initial characterization, crystals were observed to undergo a unit-cell contraction compared with the 295 K sample of 9.1% when cryocooled, 8.8% when wicked and cryocooled and 12.1% when dehydrated to a RH of 91% and cryocooled (Fig. 3 ▸, Supplementary Table S2). X-ray diffraction was observed to a resolution of 2.5–3 Å and the mosaicity was lowest in the 295 K crystals.

Dehydration followed by cryocooling showed that the unit-cell volume can decrease by up to 3.3% with respect to the cryocooled sample. Dehydration and 295 K data collection resulted in a unit-cell volume decrease of up to 7% with respect to the initial 295 K crystal (Fig. 4 ▸, Supplementary Table S2). The crystal dehydrated at 295 K had a starting unit-cell volume that was 6.3% smaller than the initial 295 K crystal and only contacted by a further 0.7% during the dehydration process.

When undertaking a HC1 experiment it is important to always characterize the crystals being worked with. When trying to deconvolute an experiment across a number of crystals over a period of days or weeks, crystal-to-crystal variation is problematic. By using larger sample sizes, *i.e.* tens of crystals at each dehydration point, this variation can be overcome.

#### 3.2.4. JMJD2D   

The *P*4 JMJD2D crystals were observed to undergo a unit-cell contraction compared with the 295 K sample of 5.2% when cryocooled, 8.3% when wicked and cryocooled and 11.6% when dehydrated to a RH of 91% and cryocooled. However, dehydration triggered an increase in the variability of the crystals, as indicated by the increase in the standard deviation of the *a* and *b* unit-cell axes from less than 1 to 3.6 (Fig. 3 ▸, Supplementary Table S3). X-ray diffraction was observed to a resolution of better than 2 Å and the mosaicity was lowest in the 295 K crystals.

When the crystals were dehydrated and cryocooled, a maximum of 26.5% unit-cell contraction was observed with respect to the initial cryocooled sample. However, there are very few reflections indexed on a small number of images at the maximum dehydration and caution is required in interpreting this result. Additionally, the standard deviation of the *a* and *b* unit-cell axes between samples is high (4–5.3) owing to large crystal-to-crystal variability (Fig. 4 ▸, Supplementary Table S3). This highlights the fact that while the unit cell is capable of change, this change correlates with an increase in the sample variability. Concomitantly, there is a decrease in the data quality as assessed by the number of indexable reflections and the data resolution, which decreased to 10–15 Å.

Dehydration and data collection at 295 K gave a different result. The data quality remained consistent, but de­hydration resulted in a unit-cell contraction of only 2.0% (Fig. 4 ▸, Supplementary Table S3). These results suggest that it is the act of cryocooling the dehydrated sample that forces the dehydrated crystal to contract and that dehydration alone does not trigger this change. Characterizing the crystal in this way highlights that future work could be carried out at 295 K without dehydration where the crystals are stable and give low-mosaicity, high-resolution data. The dramatic decrease in data quality indicates that while dehydration can change the unit cell, the crystal quality is destroyed in the process.

#### 3.2.5. MINA53   

MINA53 crystals were grown in space group *P*23. Crystals were observed to undergo a unit-cell contraction compared with the 295 K sample of 3.6% when cryocooled, 0.9% when wicked and cryocooled and 6.1% when dehydrated to a RH of 91% and cryocooled. The latter result is supported by the results of dehydration and cryocooling, where a maximum unit-cell volume decrease of 3.5% with respect to the cryocooled unit cell was observed (Fig. 3 ▸, Supplementary Table S4). In this case dehydration to a RH of 99 and 98% was tolerated, but at 96% the diffraction images could not be indexed. At a RH of 94 and 92% the diffraction images were indexable again, followed by no results on dehydration to a RH of 70%. This demonstrates that the crystals undergo a transition during dehydration. The mosaicity measurements support the transition model: the mosaicity (which is lower in cryocooled crystals that those at 295 K during the initial characterization) reaches a minimum at a RH of 92% (Fig. 4 ▸, Supplementary Table S4).

Similarly to JMJD2D, MINA53 crystals showed no lattice change when dehydrated at 295 K (Fig. 4 ▸, Supplementary Table S4). This experiment, and that of JMJD2D above (§[Sec sec3.2.3]3.2.3), highlight the importance of collecting data from dehydrated crystals at both 295 and 100 K. While in these cases the 100 K data collections showed susceptibility to dehydration, it has also been observed that dehydrated crystals result in good diffraction at 295 K and no diffraction when cryocooled (data not shown).

#### 3.2.6. PHIP(2)   

PHIP(2) crystallized in space group *P*3. The crystals were observed to undergo a unit-cell contraction compared with the 295 K sample of 14.9% when cryocooled and 14.9% when wicked and cryocooled. No diffraction was observed when crystals were dehydrated to a RH of 91% and cryocooled. Interestingly, the *a* and *b* axes only contracted by 1 Å but the *c* axis changed by 11 Å, causing the large change in unit-cell volume (Fig. 3 ▸, Supplementary Table S5). However, in the initial characterization, the cryocooled dehydration experiment and the 295 K dehydration experiment it was not possible to index images from dehydrated crystals, indicating that dehydration is not tolerated by these samples (Fig. 4 ▸, Supplementary Table S5).

#### 3.2.7. TPH2   

The *P*222 TPH2 crystals diffracted weakly throughout the study, with a maximum resolution of 7.5 Å. The crystals were observed to undergo a unit-cell contraction compared with the 295 K sample of 8.5% when cryocooled, 4.1% when wicked and cryocooled and 10.1% when dehydrated to a RH of 91% and cryocooled (Fig. 3 ▸, Supplementary Table S6). The mosaicity was lowest in the cryocooled crystals where no wicking had been applied.

The maximum extent of lattice contraction was observed at a RH of 92% and was 14% with respect to the initial 295 K crystal (Fig. 4 ▸, Supplementary Table S6). However, during the dehydration the already weak diffraction only became weaker, with diffraction to worse than 10 Å resolution.

#### 3.2.8. Proteinase K   

In the initial characterization of proteinase K the *P*4 unit cell expanded by 0.7% on cryocooling. However, the crystals were observed to undergo a unit-cell contraction compared with the 295 K sample of 2.5% when wicked and cryocooled and 4.6% when dehydrated to a RH of 91% and cryocooled (Fig. 3 ▸, Supplementary Table S7). These results indicate that the cryoprotectant used in these experiments was not ideal for this protein crystal. The crystals diffracted to 2.5 Å resolution or better and the mosaicity was lower in the 295 K crystals than those that were cryocooled.

With dehydration followed by cryocooling the maximum unit-cell contraction was 7.1% with respect to the cryocooled unit cell. With dehydration at 295 K the maximum contraction was 2.5% with respect to the 295 K cell. While the diffraction throughout was strong, it should be noted that the quality of the data did decrease with dehydration, giving rise to both higher mosaicity and a resolution of below 4 Å.

A review of the proteinase K structures available in the PDB highlights that there are two unit-cell populations which can be distinguished by the length of the *c* axis. Between the two populations there are two cases with an intermediate *c* axis. In the crystals discussed here, the cryocooled unit cell observed in the initial characterization falls into the intermediate population and all other crystals fall into the population with a small *c* axis. This shows that the crystals in this case already have the smallest successful unit cell and consequently cannot rearrange to a smaller cell upon dehydration.

#### 3.2.9. Glucose isomerase   

The initial characterization of glucose isomerase immediately highlighted an interesting case. Crystals were found to belong to either space group *P*222 or *I*222 when indexed. The *I*222 crystals were observed to undergo a unit-cell contraction compared with the 295 K sample of 4.1% when cryocooled, 3.6% when wicked and cryocooled and 3.6% when dehydrated to a RH of 91% and cryocooled. In the initial characterization no example of 295 K *P*222 crystals were seen. The cryocooled and wicked crystals differed by only 0.6% and the dehydrated and cryocooled crystals had contracted by 3.7%. The crystals diffracted to 2 Å resolution or better and the mosaicity was lower in the 295 K crystals than those that were cryocooled.

The generic protocol dehydration was applied and again identified both *P*222 and *I*222 glucose isomerase crystals. It was unclear what triggered the two point groups and this target was marked out for an extended dehydration study.

### 3.3. Extended dehydration study of glucose isomerase   

The generic dehydration protocol clearly identified that the glucose isomerase samples were changing dramatically as a result of dehydration. Having observed two potential point groups (§[Sec sec3.2.9]3.2.9), an extended study was carried out to further investigate the impact of the hydration state on the crystal lattice. For this work 14-day-old glucose isomerase crystals were used and the RHi of the mother liquor was empirically determined to be 96%.

The RH at which dehydration triggers a change in space group was tested and identified. Dehydration to a RH of 85% or lower triggers the space-group change from *I*222 to *P*2_1_2_1_2, and dehydration to a RH of 90% or higher does not impact the lattice (Table 1 ▸). Between a RH of 85% and a RH of 90% the data become difficult to index accurately while the crystal is changing (Table 1 ▸). During this transition data can be indexed in *I*222 or *P*2_1_2_1_2, but whilst these intermediate structures have been solved, the electron-density maps correlate poorly with the structures. This highlights the importance of critically examining all electron-density maps obtained after dehydration to ensure that they correspond to truly homogenous data. To optimize the data homogeneity a further dehydration step may be necessary or a longer incubation in the dehydrated humid airstream may be required.

Testing the rate of dehydration confirmed that dehydration at 1% per minute, 2% per minute, 5% per minute, 8% per minute (the HC1b maximum for this range) and abruptly by mounting the crystal in a dehydrated airstream below 85% all trigger the same space-group change (Table 1 ▸) from *I*222 to *P*2_1_2_1_2.

In order to understand the changes dehydration causes to the crystal lattice and to confirm the robustness of the method, three crystals were mounted at RHi and full data sets were first collected at 295 K and subsequently at 100 K from each crystal. In all three cases the *I*222 space group was observed at both 295 and 100 K. Cryocooling the crystal had only a small impact on the unit-cell volume, resulting in a 2.6% unit-cell volume decrease with respect to the 295 K data. Another three crystals were mounted directly into a stream of air at a RH of 70% and data were collected at 295 K and subsequently at 100 K from each crystal. In all three cases the *P*2_1_2_1_2 space group was observed at both 295 and 100 K, and cryocooling the crystal caused a unit-cell volume decrease of 4.5%. The structures of the 295 and 100 K crystals at RH 96% and RH 70% have been solved (Table 3 ▸).

During these experiments, it was observed that insufficient wicking of the sample provides a protective layer around the crystal. This layer must first be dried from the crystal before dehydration can impact the sample. The reverse is also true: exposing the crystal to dry air during manipulations will allow uncontrolled dehydration prior to the experiment. In both cases this can generate misleading results. This sample-to-sample variation makes it important to test multiple samples before drawing any conclusions.

**Figure 5:**
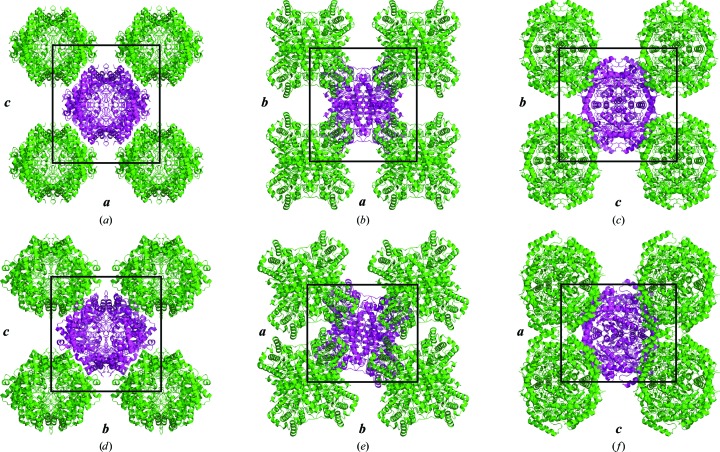
Glucose isomerase crystal lattice changes upon dehydration. Cartoon diagrams of the *I*222 hydrated (*a*, *b*, *c*) and *P*2_1_2_1_2 dehydrated (*d*, *e*, *f*) structures of glucose isomerase. The central tetramer (magenta) is the biological molecule and four surrounding tetramers are shown for each structure. The three columns show the views down each of the equivalent crystallographic axes: (*a*, *d*) *I*222 *b* axis, (*b*, *e*) *I*222 *c* axis, (*c*, *f*) *I*222 *a* axis. The *a*, *b* and *c* axes are labelled . This figure was prepared using *PyMOL* (Schrödinger).

To better understand the effect of dehydration on the crystal packing, the structures of the *I*222 and *P*2_1_2_1_2 isoforms of glucose isomerase were superposed using the first chain of each structure. The C^α^ backbones from the two structures have an overall root-mean-squared deviation (r.m.s.d.) of 0.412 as calculated using *LSQ Superpose* implemented in *Coot*. This indicates that any changes induced by dehydration occur globally, *i.e.* within the lattice, rather than at the individual monomer level. To demonstrate this, Fig. 5 ▸ shows the structures of both the *I*222 and *P*2_1_2_1_2 isoforms of glucose isomerase with the biological tetramer in the centre of each panel and four neighbouring tetramers surrounding it.

As a result of dehydration, the original tetramers come closer together along the *I*222 crystallographic *b* axis by 30 Å (Figs. 5 ▸ *a* and 5 ▸ *d*) and, as a result of the new crystal contacts, the molecules undergo a rotation about the *c* axis by 12° (Figs. 5 ▸ *b* and 5 ▸ *e*). The rotation induces the appearance of screw axes along both the *a* and *b* axes of the *I*222 lattice, giving rise to the *P*2_1_2_1_2 lattice. Owing to the indexing routines, it transpires that the naming of these axes becomes swapped. The original *I*222 *a* axis is equivalent to the new *P*2_1_2_1_2 *b* axis and *vice versa*. These axes, despite both being orthorhombic, are not directly equivalent. The rotation of the tetramers means the lattice face defined by the *a* and *b* axes of the *P*2_1_2_1_2 space group is rotated 12° with respect to the original *I*222 *ab* face. Along the *I*222 *a* axis the molecules spread out and the cell expands from 94 to 95 Å, but along the *b* axis the unit-cell dimension reduces by 16 Å. The third dimension is truly equivalent, despite the twist in the other directions, and the molecules only undergo a small translation (Table 3 ▸). As already shown by the indexing values, the molecules come closer and the *c* dimension shrinks from 103 to 98 Å. These results highlight the care that is needed when interpreting the partial results available during the initial phases of dehydration experiments and/or during the initial stages of crystal screening. Often researchers assume that slight indexing differences are subtle changes in the lattice that they are studying, but they may be missing considerable lattice shifts masked by the numerical parameters of the indexing results.

A survey of previous glucose isomerase structures shows that most structures in the PDB are of the hydrated *I*222 state. The *P*2_1_2_1_2 dehydrated form presented here might have appeared in previous work by Dauter *et al.* (1989 ▸). In this early work, which was undertaken using samples in capillaries at 295 K, three forms of *I*222 crystals were described. Two of them, defined as forms *A* and *C*, correspond to hydrated structures. The dimensions of the *B* form suggest this could be a partially dehydrated form in which the *c* cell dimension (87 Å) is probably equivalent to the *b* axis presented in this work and may be in an intermediate hydration state.

More recent work focused on sulfur SAD phasing using glucose isomerase (Ramagopal *et al.*, 2003 ▸) crystallized in the presence of manganese, as in the structures presented here, shows the structure and lattice arrangement of a *P*2_1_2_1_2 form of glucose isomerase. The *P*2_1_2_1_2 data set was collected from an older batch of crystals and was a chance finding (Z. Dauter, personal communication). At a first glance the two *P*2_1_2_1_2 structures might be assumed to be the same, with age leading to dehydration. However, careful analysis shows that the two transitions are different. In the case of the structures presented in Ramagopal and coworkers the equivalent lattices in the *I*222 structure are not identical to those presented here. In fact, in the transition from *I*222 to *P*2_1_2_1_2 presented by Ramagopal and coworkers the *a*, *b* and *c* axes of the *I*222 structure are equivalent to the *c*, *a* and *b* axes in the *P*2_1_2_1_2 structure, respectively. If this was caused by dehydration from the *I*222 structure then the key contraction is along the *a* axis (rather than the *b* axis as presented above). Rotation is induced along the *a* axis by 24° (rather than 12° along the *c* axis) and the *b* axis has been stretched to give the new structure. It should be noted that it is speculation that the results observed by Ramagopal and coworkers are a result of dehydration owing to the age of the crystal. However, it does serve as an example in which care is needed in handling crystals and collecting and interpreting the crystallographic data.

These observations, coupled with our own studies (not presented), suggest that the age of the crystal may be important in the dehydration experiment. This should be taken into account when preparing an extended study of samples, where, in order to maintain consistent results, the researcher should aim to use crystals of a similar age throughout the full experiment. Additionally, it provides a reminder to screen old plates routinely when looking for improved diffraction, since this may provide a simpler solution than finding new crystallization conditions.

## 4. Conclusions   

Nine crystal systems have been investigated using a generic dehydration protocol to assess the changes induced by crystal dehydration. The initial crystal characterization, which is the first step in the generic protocol, was an efficient analysis of the crystal. Data were collected at 295 K, with crystals cooled to 100 K using standard cryoprotection, with naked crystals cooled to 100 K and with crystals following a one-step dehydration to a RH of 91% and cooled to 100 K. The data presented above (§[Sec sec3.2]3.2) provide an evidence base for testing crystals in a number of ways prior to discarding them in favour of finding a new crystal type. Firstly, they support the collection of 295 K data from all crystals. In seven of the eight indexable examples presented, the mosaicity of the crystals was lower at 295 K than after manipulation and cryocooling. Secondly, they provide a test set of samples for which the flash-cooling of a naked crystal without dehydration was sufficient to prevent the formation of crystalline ice in the sample and was therefore a suitable alternative to chemical cryoprotection, as observed in Pellegrini *et al.* (2011 ▸). Thirdly, we demonstrate that cryocooling the crystal in the presence of an appropriate cryoprotectant causes a thermal contraction of the crystal in seven of the eight indexable systems. Additionally, in every case presented flash-cooling the naked crystal in the absence of dehydration caused a contraction of the unit cell.

In five of the nine systems, glucose isomerase, TPH2, Tudor, JMJD2D and MINA53, changes to the unit cell or point group were seen during dehydration. In the remaining four systems either no change was observed or the change was so dramatic that no indexable diffraction could be measured. 56% of this test set of crystallization systems demonstrated change in the unit cell, which is a prerequisite for improving X-ray diffraction using the HC1b. However, in this set there is not an example where improved diffraction resolution was observed.

With only two exceptions, TPH2 and PHIP(2), we observe that diffraction at 295 K gives a lower mosaicity than that observed at 100 K, independent of the method of cryocooling. Consequently, it is recommended that all crystals are tested at 295 K to investigate their potential for diffraction. The HC1b provides a convenient route to testing crystals at 295 K by using pins and harvesting techniques familiar to those already using cryocrystallography.

Dehydration causes a change in the crystal lattice away from the initial ordered state. This is reflected by an increase in mosaicity and a more disordered diffraction pattern. Only if a second ordered state can be achieved will the diffraction pattern improve and the mosaicity decrease.

In eight of our nine systems it was possible to successfully cryocool naked crystals in the absence of a chemical cryoprotectant. It has been observed that the background noise in diffraction data collected from naked crystals is lower than from their chemically cryoprotected counterparts (Pellegrini *et al.*, 2011 ▸). Consequently, when optimizing crystal cryoprotection it would be wise to include tests of the naked crystals alongside crystals prepared with the range of available chemical cryoprotectants.

It is notable that in the cases of JMJD2D and MINA53 the unit-cell contraction was twelve and nine times larger, respectively, in crystals prepared and tested at 100 K in comparison to crystals at 295 K. By contrast, the Tudor protein crystals contracted nearly twice as much at 295 K as at 100 K. These observations imply that cryocooling a dehydrated crystal may impact a unit-cell change that occurs during dehydration. These three examples demonstrate that a difference in behaviour can be observed between crystals at 295 and at 100 K and that a full investigation requires both experiments.

Ultimately, the data provided here show that the complete dehydration experiment should pursue a wholly 295 K experiment and a laboratory-based 100 K study, as well as extended investigations where indicated. By adopting the approach described in Fig. 1 ▸, glucose isomerase was identified as a system of interest for the dehydration experiment and it has been possible to identify the nature of the changes induced by dehydration through the two structures presented.

The HC1b is available both on the beamline for 295 K studies and also in the Diamond Light Source laboratory facility, enabling users to explore a large number of relative humidity conditions on multiple crystals without disrupting the high-throughput nature of MX beamtime. Finally, it is clear that dehydration affects every crystal system studied differently and it is only by investigating these effects that a scientist can determine how useful dehydration will be for their specific crystals.

## Supplementary Material

PDB reference: glucose isomerase, native, room temperature, 4zb2

PDB reference: dehydrated, room temperature, 4zb0

PDB reference: native, 100 K, 4zb5

PDB reference: dehydrated, 100 K, 4zbc

## Figures and Tables

**Table 1:** Extended glucose isomerase study

Starting humidity (%)	Dehydration rate (% per minute)	Final humidity (%)	Crystal class
96	0	96	*I*222
96	1	64	*P*222
96	2	64	*P*222
96	5	64	*P*222
96	8 (machine-limited)	70	*P*222
90	0	90	*I*222
88	0	88	*I*222/*P*222
85	0	85	*P*222
80	0	80	*P*222
70	0	70	*P*222
80	2	70	*P*222
80	5	70	*P*222

**Table 2:** Relative humidity of crystallization mother liquor

Protein	Primary precipitant	Predicted RHi† (%)	Determined RHi (%)
JMJD1B	27% PEG 3350	98.6	98
BAZ2B	36% PEG 600, 8% glycerol	94.5	94
Tudor	1.6–2 *M* ammonium sulfate	96.8	97
JMJD2D	26% PEG 3350	98.6	99.9
MINA53	18% PEG 3350	99.4	99.9
PHIP(2)	6% PEG 3350	99.9	99.9
TPH2	14% PEG 3350, 10% ethylene glycol	95.6	96
Proteinase K	20% PEG 3350	99.3	99.9
Glucose isomerase	20–30% glucose‡, 10% PEG 400	95–96.5	94–96

† Predicted values were determined using the Java application at http://www.embl.fr/CrystalDehydrationCollaboration/RH.html (Wheeler *et al.*, 2012 ▸).

‡ While glucose is not strictly a precipitant, it is acting as a precipitant in this situation and impacts the RH of the solution.

**Table 3:** X-ray data-collection and structure-refinement statistics for glucose isomerase

	*I*222, 295 K	*I*222, 100 K	*P*2_1_2_1_2, 295 K	*P*2_1_2_1_2, 100 K
Data collection				
X-ray source	I02	I02	I02	I02
Wavelength (Å)	0.9795	0.9795	0.9795	0.9795
Space group	*I*222	*I*222	*P*2_1_2_1_2	*P*2_1_2_1_2
Unit-cell parameters (Å, °)	*a* = 94.07, *b* = 99.22, *c* = 103.03, α = β = γ = 90	*a* = 92.90, *b* = 98.15, *c* = 102.70, α = β = γ = 90	*a* = 83.49, *b* = 95.02, *c* = 98.48, α = β = γ = 90	*a* = 81.63, *b* = 93.64, *c* = 97.64, α = β = γ = 90
Resolution (Å)	2.0	2.0	2.0	2.0
Mosaicity (°)	0.15	0.55	1.1	1.1
*R*_merge_	0.051 (0.086)	0.050 (0.068)	0.060 (0.138)	0.044 (0.103)
〈*I*〉/σ(〈*I*〉)	18.0 (12.9)	18.7 (15.4)	10.4 (5.9)	14.4 (8.6)
Completeness (%)	99.8 (99.7)	99.8 (99.8)	99.5 (99.6)	99.5 (99.8)
Multiplicity	4.5 (4.6)	4.4 (4.6)	3.2 (3.3)	3.2 (3.3)
Refinement				
No. of unique reflections	30438	31979	50546	50965
*R*_cryst_	0.106	0.116	0.137	0.136
*R*_free_	0.140	0.156	0.180	0.182
No. of residues				
Protein	387	388	776	776
Ligands	4	3	14	19
Water	301	518	446	542
Average *B* factors (Å^2^)				
Protein	17.86	8.54	20.38	14.18
Sugar	18.90	6.82	20.98	30.49
Manganese	11.00	6.44	34.46	28.92
Waters	29.67	23.51	33.00	26.22
R.m.s. deviations				
Bond lengths (Å)	0.021	0.023	0.022	0.018
Bond angles (°)	2.077	2.011	2.371	2.029
Ramachandran statistics (%)				
Favoured	97.1	97.1	96.5	97.0
Allowed	2.6	2.6	3.2	2.7
Outliers	0.3	0.3	0.3	0.3
PDB code	4zb5	4zb2	4zb0	4zbc
